# Proton mediated control of biochemical reactions with bioelectronic pH modulation

**DOI:** 10.1038/srep24080

**Published:** 2016-04-07

**Authors:** Yingxin Deng, Takeo Miyake, Scott Keene, Erik E. Josberger, Marco Rolandi

**Affiliations:** 1 Department of Materials Science and Engineering, University of Washington, Seattle, WA 98195-2120, USA; 2 Department of Electrical Engineering, Jack Baskin School of Engineering, University of California, Santa Cruz, CA 95064, USA; 3 Department of Electrical Engineering, University of Washington, Seattle, WA 98195-2500, USA

## Abstract

In Nature, protons (H^+^) can mediate metabolic process through enzymatic reactions. Examples include glucose oxidation with glucose dehydrogenase to regulate blood glucose level, alcohol dissolution into carboxylic acid through alcohol dehydrogenase, and voltage-regulated H^+^ channels activating bioluminescence in firefly and jellyfish. Artificial devices that control H^+^ currents and H^+^ concentration (pH) are able to actively influence biochemical processes. Here, we demonstrate a biotransducer that monitors and actively regulates pH-responsive enzymatic reactions by monitoring and controlling the flow of H^+^ between PdH_x_ contacts and solution. The present transducer records bistable pH modulation from an “enzymatic flip-flop” circuit that comprises glucose dehydrogenase and alcohol dehydrogenase. The transducer also controls bioluminescence from firefly luciferase by affecting solution pH.

In nature, H^+^ plays an important role in modulating enzymatic reactions during metabolic process to fulfill physiological functions^1^,^2^. Examples include glucose oxidation with glucose dehydrogenase to maintain the blood glucose level, alcohol digestion into carboxylic acid through alcohol dehydrogenase, and voltage-regulated H^+^ channels activating bioluminescence in firefly and jellyfish^3^. There are several bioelectronics devices that use enzymatic reactions to regulate device output such as biofuel cells^4^, organic electrochemical transistors biosensors^5^, and enzyme logics mimicking electronic circuits^6^. In turn, bioelectronic devices may regulate electron mediated enzymatic reactions^7^,^8^. However, not all enzymatic reactions are electron mediated and control of enzymatic reactions with devices^9^,^10^,^11^,^12^ that modulate ionic currents is desirable.

Examples of these devices include ion pumps based on conducting polymers that can deliver Ca^2+^ and the positive charged neurotransmitter gamma-aminobutyric to brain tissues for potential treatment of epilepsy^13^, metallic nanostraws^14^ and carbon nanotube porins that^15^,^16^ deliver selectively target molecules such as cations^15^, DNA^14^, and nicotine^16^. H^+^ also plays important roles in modulating physiological function^17^. We have developed bioprotonic devices that selectively control the flow of H^+^ including complementary field effect transistors^18^,^19^, synaptic memories^20^, and enzyme logic^6^. Recently, H^+^ transistors with the protein reflectin have been demonstrated^21^. These bioprotonic devices are based on PdH_x_ as a transducer between H^+^ currents in the device or solution and e^−^ current in the electronic circuit. Here, we develop an electrochemically-controlled PdH_x_ biotransducer that not only records but also modulates the pH of a solution. We integrate this biotransducer with enzymatic reactions to implement H^+^ mediated monitoring and control of biochemical processes (Fig. 1). The biotransducer converts the input of an enzymatic flip-flop circuit – a bistable circuit with a high pH and a low pH state controlled by the enzymes glucose dehydrogenase and alcohol dehydrogenase- to a readable H+ current as the output (Fig. 1a), and it provides pH modulation as the input for the pH sensitive enzymatic reaction of luciferin and luciferase with bioluminescence as the output (Fig. 1b).

## Results

PdH_x_ protodes measure the pH of a solution because the transfer of H^+^ between PdH_x_ and the solution is affected by the difference in chemical potential of H^+^, or protochemical potential (μ), between the PdH (μ_PdH_) and the solution (μ_pH_) (Fig. S1)^6^,^22^. This difference is defined as^22^


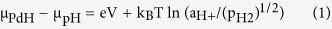


where a_H+_ = activity of H^+^ in solution with pH = −log a_H+_, p_H2_ = hydrogen partial pressure in the Pd V = potential difference between Pd and solution

**Figure 1:**
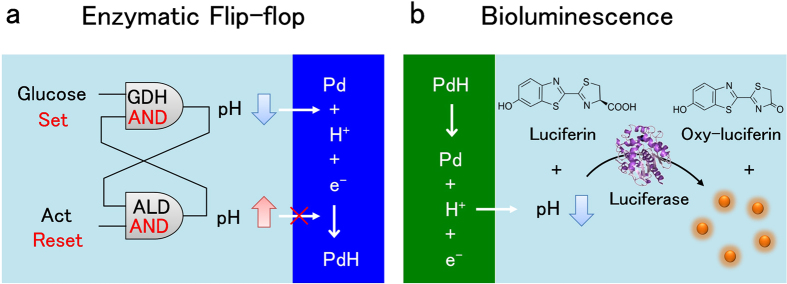
H^+^ mediated enzymatic reactions. (**a**) Schematic of the enzymatic flip flop circuit as a bistable pH modulating system controlled by logically processed biochemical signals. The ‘set’ operation is the glucose input. The reaction between glucose and the enzyme glucose dehydrogenase (GDH) increases H^+^ concentration in solution and lowers the pH. Acetaldehyde (Act) reacts with the alcohol dehydrogenase (ALD) enzyme. The reaction consumes H^+^ in the reaction and increases the pH. The GDH and ALD AND enzyme logics regulate the solution pH, thus affect H^+^ transfer between solution and the contact. H^+^ combines with an electron at the Pd contact and forms PdH. The PdH biotransducer converts the biochemical signals into readable protonic current. (**b**) Schematic of firefly bioluminescence reaction integrated with pH modulating biotransducer. Luciferin is oxidized into oxy-luciferin with the presence of the luciferase enzyme. The reaction emits light. The solution pH affects the color of the light emitted. When the PdH pH modulator transfers H^+^ between the solution and the contact, it changes the solution pH. The bioluminescence light is the solution pH change readout.

According to PdH ↔ Pd + H^+^ + e^−^, the H^+^ transfer results in a measurable electronic current. In our previous work, we have demonstrated recording of the pH readout of an enzymatic AND gate comprising glucose (Glc) and NAD^+^ with glucose dehydrogenase (GDH)^15^. In the presence of both NAD^+^ and Glc, GDH lowers the solution pH, which is recorded with the PdH_x_ protodes. In the absence of either substrate, GDH is not active and the solution pH remains the same^23^. GDH modifies the pH only once because the enzymatic AND gate does not include a feedback loop to make the reaction reversible and return the solution to its original pH. Here, we integrate the enzyme alcohol dehydrogenase (ALD) with GDH to create an enzymatic flip-flop with a bistable pH modulation as the read out (Fig. 1a). A flip- flop is a circuit made by two AND gates. The output of the first AND gate is fed back as one of the inputs of the second AND gate and vice versa. In this fashion, a flip-flop has two stable states and can be used to store information. A set and a reset inputs switch the flip-flop between the two states. The enzymatic flip-flop consists of two types of enzymatic AND logic that use GDH and ALD as gates. GDH requires the presence of both NAD^+^ and Glc as inputs to function. GDH produces NADH and gluconic acid (GlcA), which lowers the solution pH from 6.0 to 4.3 (Fig. S2a). NADH, the output of the GDH, is one of the inputs for ALD with acetaldehyde (Act) as the other input. In the presence of both NADH and Act, ALD produces ethanol and NAD^+^. NAD^+^, in turn, is the input of the GDH logic gate. ALD consumes H^+^ during this enzymatic reaction and induces an increase in solution pH from 7 to 9.7 (Fig. S2b). The products (NADH or NAD^+^) of each AND gate (ALD or GDH) are connected to one of the inputs of the other gate. This connection results in a positive feedback that creates an enzymatic flip-flop^24^. The life-time of the flip-flop circuit depends on the life-time of the enzyme activity. While extended lifetime is not the goal of the present work, we measure at least three cycles of the bistable pH modulation and the devices are functional for at least one day when stored at low temperature. In our system, the ‘set’ operation is Glc injection, which causes a decrease in pH and an associated increase in the solution μ_pH_^6^. The reset operation is Act injection, which causes an increase in pH and an associated decrease in the solution μ_pH_. Solution pH is used as the readout.

**Figure 2:**
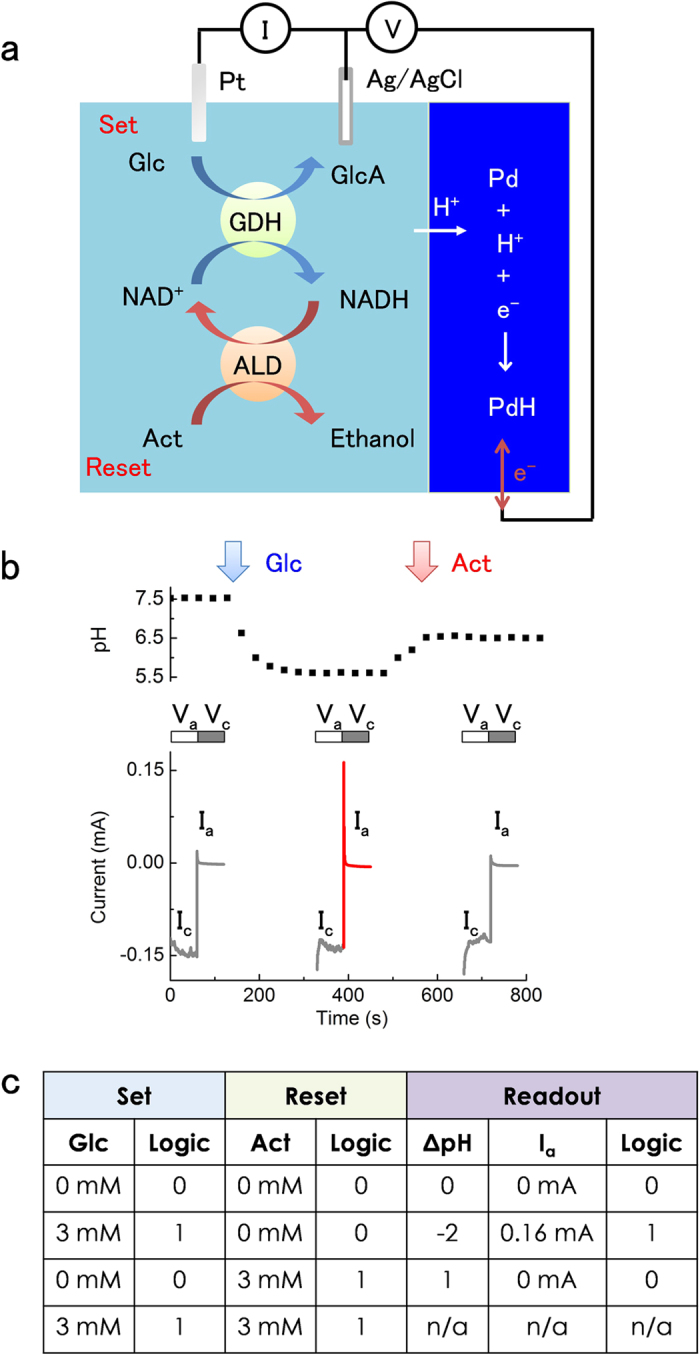
The biotransducer transduces the set-reset enzymatic flip-flop signals into current. (**a**) Enzymatic flip-flop integrated with the PdH biotransducer. The electrochemical setup has a Pd working electrode, Ag/AgCl reference electrode and Pt counter electrode. V_a_ and V_c_ is applied to Pd vs. Ag/AgCl. I_a_ and I_c_ is measured between Pd and Pt. The reaction between glucose and NAD^+^, catalyzed by the enzyme GDH, produces gluconic acid (GlcA) and NADH. Acetaldehyde (Act) reacts with the ALD enzyme and produces ethanol and NAD^+^. (**b**) Upper panel shows the pH change when adding Glc and Act into the solution. Lower panel shows I_c_ and I_a_ in response to pH change. V_c_ = 0 V and V_a_ = −0.95 V is applied during the ‘set’ and ‘reset’ process respectively. I_c_ is recorded during V_c_ = 0 V, and I_a_ is recorded during V_a_ = −0.95 V. I_a_ is almost 0 when solution is above pH 6. At V_a_ = −0.95 V, few H^+^ can be transferred into Pd contact. pH 5.5 induced by the addition of Glc into the solution causes I_a_ = 0.16 mA. The return of pH to above 6 with the addition of Act results in no I_a_. (**c**) Truth table of the set-reset enzymatic flip-flop circuit.

To read out the state of the enzymatic flip-flop, we integrate it with the PdH_x_ electrochemical biotransducer and monitor the transfer of H^+^ between the solution and the PdH_x_ protode at a given applied voltage as function of the pH state (Fig. 2a) (Fig. S1). To this end, we use a standard three-electrode configuration with Pd (PdH_x_) as the working electrode (WE), Ag/AgCl as the reference electrode (RE), and platinum (Pt) as the counter electrode (CE). In this setup, a large enough cathodic voltage, V_c_, applied to the WE (negative vs. Ag/AgCl) transfers an H^+^ from the solution to the Pd where H^+^ combines with an e^−^ to form H. H adsorbs onto Pd to form PdH_x_. In general, H adsorption into the Pd nanofilm can expand its volume up to 10% after forming the PdH^25^, so the film thickness (50 nm Pd) will increase up to 55 nm of PdH. We monitor this H^+^ transfer by recording the cathodic current, I_c_, resulting from the e^−^ that flow into the Pd WE and participate in the reduction of H^+^ to H. A large enough reverse anodic voltage, V_a_, applied to the WE (positive vs. Ag/AgCl) causes an H^+^ to transfer from the PdH_x_ to the solution leaving an e^−^ behind, which leads to an anodic current I_a_ (electron flowing out of the Pd WE). When all of the PdH_x_ is transformed into Pd, I_a_ goes to zero. The magnitude of I_a_ is proportional to the extent of PdH_x_ formation in the cathodic phase because it is directly correlated to how many H^+^ have previously transferred from the solution into the PdH_x_^26^. We use the mangnitude of I_a_ as an indirect means of monitoring H^+^ transfer from solution into the PdH_x_ because I_c_ also contains components related to charging of the solution.

In the initial state of the flip-flop, the solution pH = 7.5 and V_c_ = −0.95 V. According to the process map (Fig. S1) H^+^ do not transfer from the solution to the Pd contact, I_c_ = 0 A, and setting V_a_ = 0 V results in I_a_ =0 A because PdH_x_ did not form when V_c_ was applied (Fig. 2b). We keep V_c_ = −0.95 V during the “set” operation when we add Glc (Fig. 2b). Adding 3 mM Glc to the solution causes the pH to drop from 7.5 to 5.5. Now, with V_c_ = −0.95 V, H^+^ transfer from the solution to the Pd contact and form PdH_x_. For PdH_x_, μ_PdH_ is higher than μ_pH_ when the pH = 5.5. When we switch to V_a_ = 0 V, H^+^ transfer back to the solution. The I_a_ resulting from this transfer is as high as 0.16 mA. The ‘reset’ operation of adding Act in the presence of NADH engages the ALD enzymatic reaction, which consumes H^+^ and brings the pH back close to neutral with a value of pH = 6.5. At pH = 6.5, H^+^ do not transfer into the Pd when V_c_ = −0.95 V and PdH_x_ does not form. As a result, when V_a_ is set to 0 V, I_a_ = 0 mA.

Using these inputs and readouts, we produce a truth table for the enzymatic flip-flop (Fig. 2c). The output, or memory readout, switches between two states defined digitally as 0 (neutral pH) and 1 (low pH). These two states correspond to either I_a_ = 0 mA or I_a_ >>0 mA. The enzymatic flip-flop is activated by ‘set’ and ‘reset’ input signals applied at two levels digitally defined as 0 and 1. The presence of the ‘set’ Glc or the ‘reset’ Act is digital 1 and their absence is digital 0. Application of ‘set’ = 0 and ‘reset’ = 0 preserve the readout state regardless of its original value (0 or 1). Application of ‘set’ = 1 results in the readout state 1, while application of ‘reset’ = 1 yields the readout state 0, regardless of the original readout state (0 or 1). As in a digital flip-flop, the simultaneous application of the signals ‘set’ = 1 and ‘reset’ = 1 sends the system in opposite directions, results in an unstable readout, and it is not permitted. The enzymatic flip-flop demonstrates how the Pd/PdH_x_ biotransducer transduces H^+^ signals in the form of pH change from the enzymatic readouts into measurable electronic currents.

**Figure 3:**
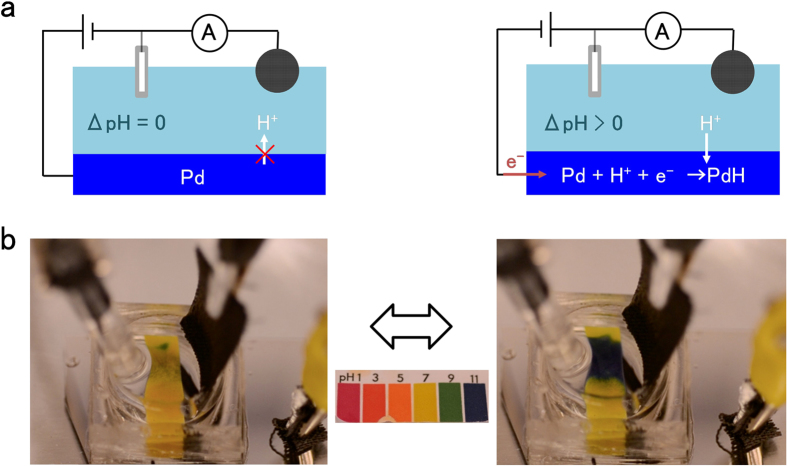
pH regulation in solution. (**a**) Schematics of pH modulator with CNT modified CF counter electrode. In (**a**), with Pd contact there is no I_a_ at V_a_ = 0.9 V. At V_c_ = −0.9 V, H^+^ transfers from solution into the Pd and recombines with e^−^ to form H. H adsorbs onto Pd and forms PdH. Decrease of H^+^ concentration in solution causes pH increase in solution. (**b**) Pictures of pH modulator with pH paper in solution. Yellow color of the paper indicates pH = 7.0 in solution, which is the initial pH of solution. Dark blue color indicates pH = 10.0 in solution. pH increase is due to H^+^ transfer from solution to Pd contact.

In addition to the protonic readout from an enzyme logic signal, we use this PdH_x_ biotransducer to modulate pH in solution (Fig. 3a). When V_c_ is applied to the WE and H^+^ transfer from solution into the Pd contact, the net concentration of H^+^ in solution decreases and the pH increases (Fig. 3a). At the same time, when V_a_ is applied to the WE and H^+^ transfer from the PdH_x_ contact into the solution, the net concentration of H^+^ in the solution increases, and the pH decreases (Fig. 3a). Since the voltage applied to the WE, V_c_ or V_a_, controls whether H^+^ flow from the solution into the PdH_x_ contacts and vice versa, the net result is electronic pH control. In order to achieve this electronic pH control, we keep the conventional three-electrode system used in enzymatic flip-flop setup. This setup, however, does not produce the desired results because of the high catalytic property of the Pt CE. It is likely that redox reactions (4H^+^ + O_2_ + 4e^−^ → 2H_2_O; 2H^+^ + 2e^−^ → H_2_; 2H_2_O + 4e^−^ → H_2_ + 2OH^−^) on the CE consume the H^+^ injected from the WE with no change in solution pH. To overcome this issue, we use a counter electrode that is inert and with high capacitance, similar to the electrodes used in electrochemical double layer capacitors^27^. We use a high capacitive carbon fabric decorated with carbon nanotube (CF/CNT), with a capacitance per unit area C_i_ = 6.5 mF/cm^2^ 28. We first attempt to increase the pH of a Na_2_SO_4_ solution with initial pH = 6.8 (Fig. 3b). With V_c_ = −0.9 V on the WE, H^+^ transfer from the solution into the WE and form PdH_x_. The H^+^ concentration in the solution decreases and the pH increases as indicated by the pH paper changing color from yellow (neutral pH) to dark blue (basic pH) (Fig. 3b). The Pd WE also changes color from metallic silver into a darker gray as expected from the transformation of Pd into PdH_x_, as we have previously observed^20^. As a control, we confirm that with an Au WE the pH change is much smaller than when using the PdH_x_ WE (Fig. S3). This is because the Au WE is not able to directly inject or sink H^+^ at the solution interface. The small pH change that we still observe with the Au WE may be due to H^+^ or OH^−^ accumulating at the Au charged surface or other surface catalytic reactions^29^. However, the pH change with PdH protode is much higher than that with Au WE because the Au does not promote H^+^ transfer between the WE and the solution. When V_a_ = 0.9 V is applied to the PdH_x_ WE, PdH_x_ injects H^+^ back into the solution, which returns to its original pH (Fig. S4). This pH modulation is reversible and solution pH cycles bewtween pH = 6.8 to pH = 9.2 several times (Fig. S5). While the inert CF/CNT enables pH modulation of solution by minimizing side reactions at the CE, likely some of these reactions remain and optimization of the system is required to increase the range of pH modulation.

**Figure 4:**
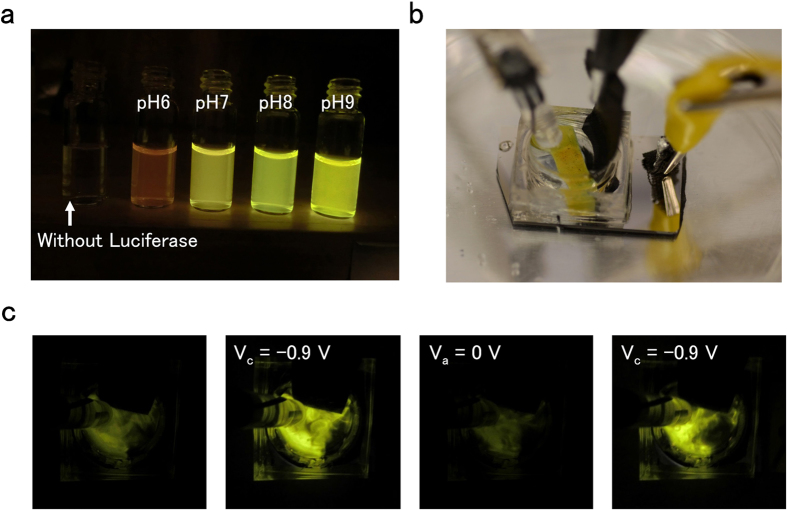
Bioluminescence incorporated with pH modulating biotransducer. (**a**) Schematic of the pH modulator integrated with bioluminescence enzymatic reaction. Change of pH lights up the solution due to the optimal pH of the enzyme reaction is reached. (**b**) The firefly bioluminescence at different solution pH. At acidic pH 6, solution shows red color. At neutral to basic pH, solution is yellow. The optimal pH of firefly luciferin luciferase reaction is pH 8, thus strongest light emission. (**c**) Bioluminescence turned on and off by the pH modulator. The initial pH of solution is 5.8. When applied V_c_ = −0.9 V, pH increases to reach the optimal pH for the enzymatic reaction. Bioluminescence turns on. When reversed to V_a_ = 0.9 V, solution pH returns to initial pH and bioluminescence is off. Bioluminescence can be turned on and off for several cycles.

We demonstrate pH control in solution with an optical output by modulating the output bioluminescent enzymatic reactions (Figs 1b and 4)^30^. We choose to modulate the yellow-green glow that in nature is emitted by the firefly. This glow is a result of the oxidation reaction of firefly luciferin, catalyzed by the enzyme luciferase in the presence of ATP as the energy source (Fig. 1b). This enzymatic reaction is pH sensitive^30^. To this end, we utilize a Japanese toy (Photolight), which contains one packet of firefly luciferase, and the other packet of its substrate luciferin, ATP powder, and Mg^2+^, which is consumed during the reaction. We test the reaction at different pH and obtain the highest output at pH = 8 (Fig. 4a). The color also changes as a function of pH from yellow to bright green and ultimately to red when the solution pH changes from basic to acidic (Fig. 4a). This color change is consistent with prior reports^31^. We place the solution at pH = 6.7 containing the Photolight components in a PDMS chamber that includes the electrode setup used previously (Fig. 4b). When V_c_ = −0.9 V at the WE, H^+^ transfer from the solution to the Pd/PdH_x_ WE and result in an increase of solution pH. When pH = 8 is reached in the solution, the bioluminescent output increases with a bright yellow color (Fig. 4c). When V_a_ = 0 V at the WE, H^+^ transfer back into the solution, which returns to its original pH turning the bioluminescence off. This pH controlled bioluminescence can be cycled between the low pH off state and the high pH on state. We have not thoroughly mapped performance and lifetime for this process. In preliminary measurements we were able to turn the bioluminescence ON and OFF for at least four cycles. (Fig. S6).

**Figure 5:**
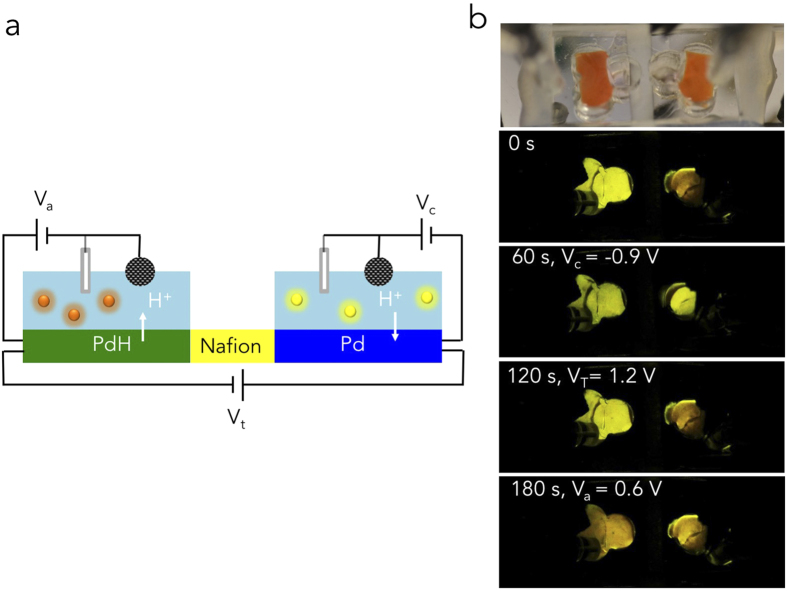
Bioluminescence incorporated with pH modulator circuit. (**a**) Schematic of the pH modulator circuit integrated with bioluminescence enzymatic reaction, (**b**) PDMS chamber contains firefly bioluminescent enzyme that changes color as function of solution pH.

To decrease solution pH in addition to increase solution pH, we develop a pH modulator circuit with two Pd/PdH_x_ WE and two separate chambers (Fig. 5a). The two chambers and Pd/PdH_x_ WE act as H^+^ reservoirs for each other. The two WE are connected by a strip of proton conducting polymer (Nafion), which transfers H^+^ between the two Pd/PdH_x_ contacts when a transfer voltage (V_T_) is applied^20^. Again, to monitor pH change in the two chambers we use firefly bioluminescence (Fig. 5b). We start with the chamber of the right at pH 6.5 and the chamber on the left at pH 7.5 as indicated by a reddish (right) and bright yellow (left) bioluminescent output. We start with the chamber on the right slightly acidic to ensure that there are enough H^+^ to transfer into the Pd WE. First, we apply V_c_ = −0.9 V to the Pd WE on the right and load it with H^+^ from the solution. Solution pH in the right chamber increases as indicated by the bioluminescent output color change from red to yellow. Second, we apply a transfer voltage V_t_ = 1.2 V between the two Pd WE to transfer the H^+^ from the right WE to the left WE along the Nafion H^+^ conducting bridge. At this stage, V_c_ = 0 V and it is likely that some of the H^+^ from the PdH move back to the solution with higher pH and lower protochemical potential^6^. This transfer results in the pH in the right chamber returning to slightly acidic. Third, we apply V_a_ = 0.6 V to the WE on the right and transfer the H^+^ from the WE to the solution in the left chamber. The pH in the left chamber decreases as indicated by a change in color from bright yellow to red. If we apply V_a_ = 0.6 V to the left chamber without previously transferring H^+^ from the right chamber and right WE, we do not observe any noticeable pH change.

## Discussion and Conclusions

We demonstrate a Pd/PdH_x_ based protonic biotransducer that records and modulates pH dependent enzymatic reactions. This recording and modulation is based on the pH dependence of the transfer of H^+^ between solution and Pd/PdH_x_. By coupling this biotransducer with two enzymatic AND gates, we demonstrate an enzymatic flip-flop that sets a high and a low pH state. Such integrated logic system may allow for future implantable biomedical devices controlled by physiological conditions. Further, we provide an optical output for pH modulation by using firefly bioluminescence with pH dependent color and intensity. While we modulate the pH of the entire solution, it is likely that the pH at the surface of the PdH_x_ contacts is different due to expected electrostatic charging effects. We are able to modulate the solution pH between 6.8 and 9.6 with an increase in efficiency required to access a broader pH spectrum. This broader pH spectrum is required for controlling biological function in solutions with high buffering capacity such as physiological conditions^26^.

## Materials and Methods

Devices are fabricated on microscope glass slide (2.5 cm × 4.5 cm VWR). 50 nm Pd with a 15 nm Cr adhesion layer is deposited via e-beam evaporation (Balzers PLS 500). PDMS wells (made from 5 mL PDMS solution) are used as solution containers and attached to the Pd substrates with PDMS. For the pH modulator circuit, 3.5 wt% agarose gel solution is used to seal between the PDMS wells and the substrates. Fully hydrated agarose gel is placed above the Nafion channel to hydrate the material. After 15 min we assume that the Nafion is fully hydrated and we begin the measurement. 200 μL Nafion 117 solution (5% concentration) from Sigma Aldrich is drop-cast on top of the patterned Pd substrate and the solution is dried in a fume hood. Glucose dehydrogenase (EC 1.1.47) is donated from TOYOBO enzymes. Alcohol dehydrogenase (EC 1.1.1.1), glucose, acetaldehyde, nicotinamide adenine dinucleotide, Na_2_SO_4_, MgSO_4_, universal pH indicator are purchased from Sigma Aldrich. Luciferin and luciferase are from the Photolight (Japan). Carbon fiber (TCC-3250) is donated from Toho Tenax Co. Carboxylic groups modified carbon nanotubes (20–30 nm) are purchased from Cheap Tubes.com. The CNT modified CF electrode follows a previously reported method^28^. Electrochemical measurements are performed with a potentiostat (BAS, model2325).

## Additional Information

**How to cite this article**: Deng, Y. *et al*. Proton mediated control of biochemical reactions with bioelectronic pH modulation. *Sci. Rep*. **6**, 24080; doi: 10.1038/srep24080 (2016).

## Supplementary Material

Supplementary Information
