# Supplementary material for: Proton mediated control of biochemical reactions with bioelectronic pH modulation
Source: Sci Rep. 2016 Apr 7;6:24080. doi: 10.1038/srep24080 (PMC4823714; doi:10.1038/srep24080)
Supplement: Supplementary Information [file srep24080-s1.pdf]

Supplementary information

Proton mediated control of biochemical reactions with bioelectronic pH modulation

Yingxin Deng,[1,†] Takeo Miyake,[1,2,†,*] Scott Keene,[1] Erik E. Josberger,[1,3] Marco Rolandi,[1,2,*]

[1]Department of Materials Science and Engineering, University of Washington, Seattle, WA 98195-2120, USA

[2] Department of Electrical Engineering, Jack Baskin School of Engineering, University of California, Santa Cruz, CA 95064, USA

[3]Department of Electrical Engineering, University of Washington, Seattle, WA 98195-2500, USA

[*]Corresponding author: mrolandi@ucsc.edu, tmiyake@ucsc.edu

[†] These authors contributed equally to this work.

Supplementary Figures

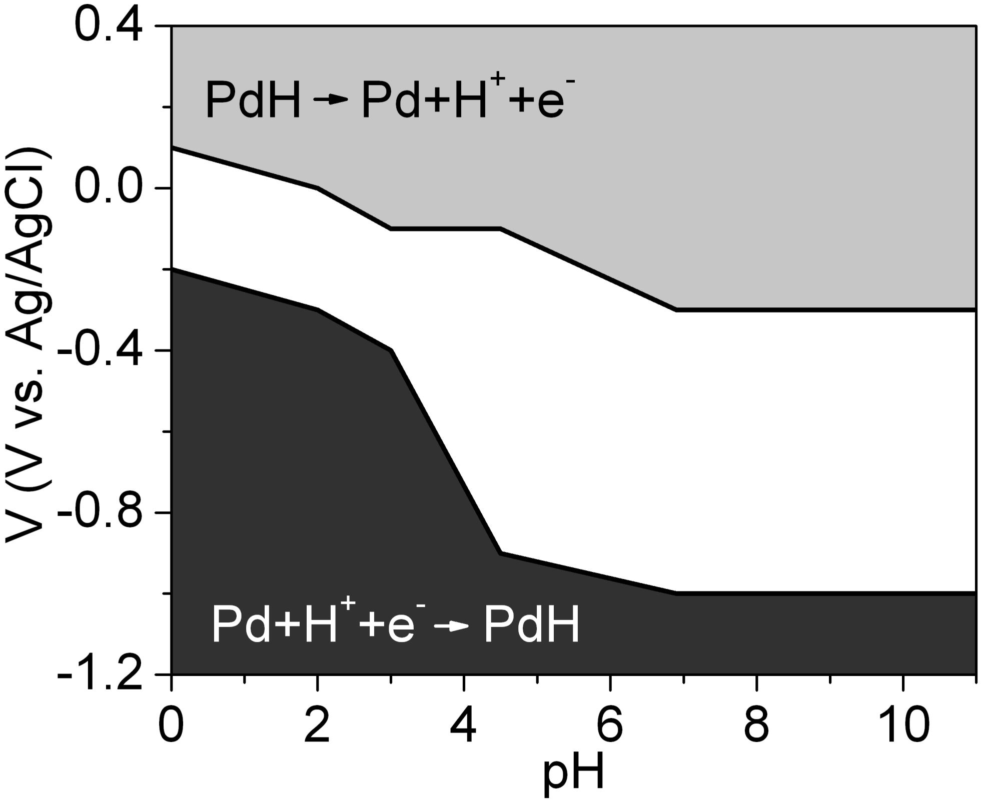

Fig. S1. Process map between voltage applied to the PdH_x working electrode and the solution pH for the reversible Pd + H^+ + e^- ←→ PdH. In the white area there is no transfer or H^+ between the solution and the Pd/PdH_x working electrode. Reproduced with permission from Applied Physics Letters Materials, 2015.

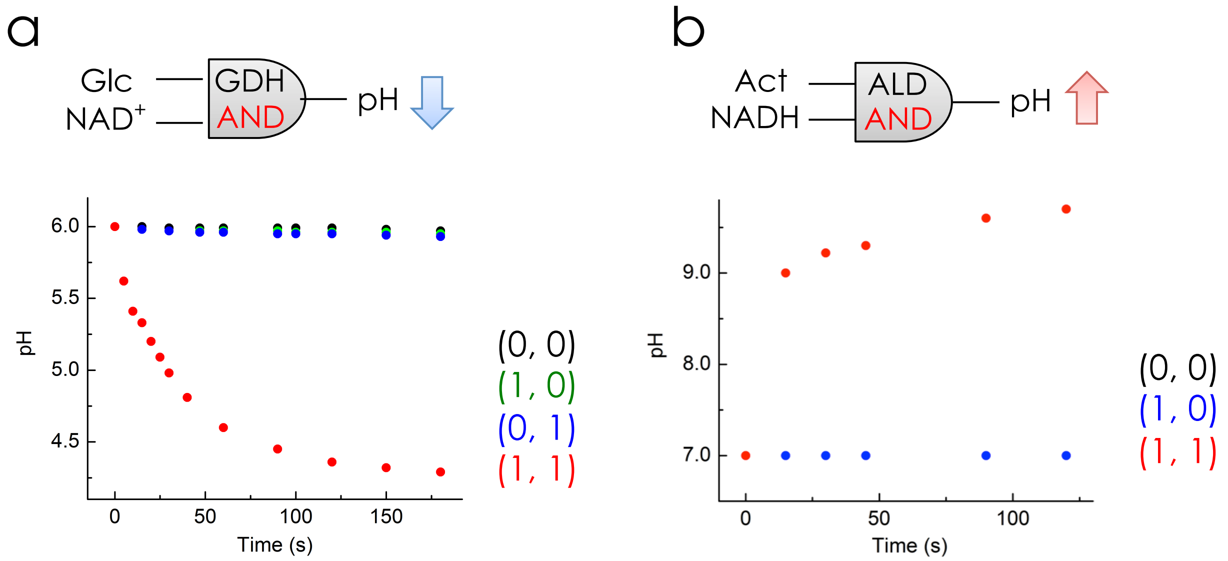

Fig. S2. (a). The GDH enzymatic reaction happens only with both Glc and NAD^+ present, similar as an AND gate. The production of gluconic acid from the reaction decreases the pH of the solution. The presence of either Glc or NAD^+ is marked as digital 1, and the absence is digital 0. The output is the pH change. The (0,0), (0,1) (1,0) input does not cause any pH change, thus output is 0. With both Glc and NAD^+ present, (1,1) input, there is a pH decrease from 6.0 to 4.3. (b). The ALD enzymatic reaction takes place only when both Act and NADH are present, similar to an AND gate. The consumption of H^+ during the reaction increases the pH of the solution. The presence of either Act or NADH is digital 1, and the absence is digital 0. The output is the pH change. The (0,0) and (1,0) input does not cause any pH change, thus output is 0, no pH change. With both Act and NADH present, (1,1) input, there is a pH increase from 7.0 to 9.7.

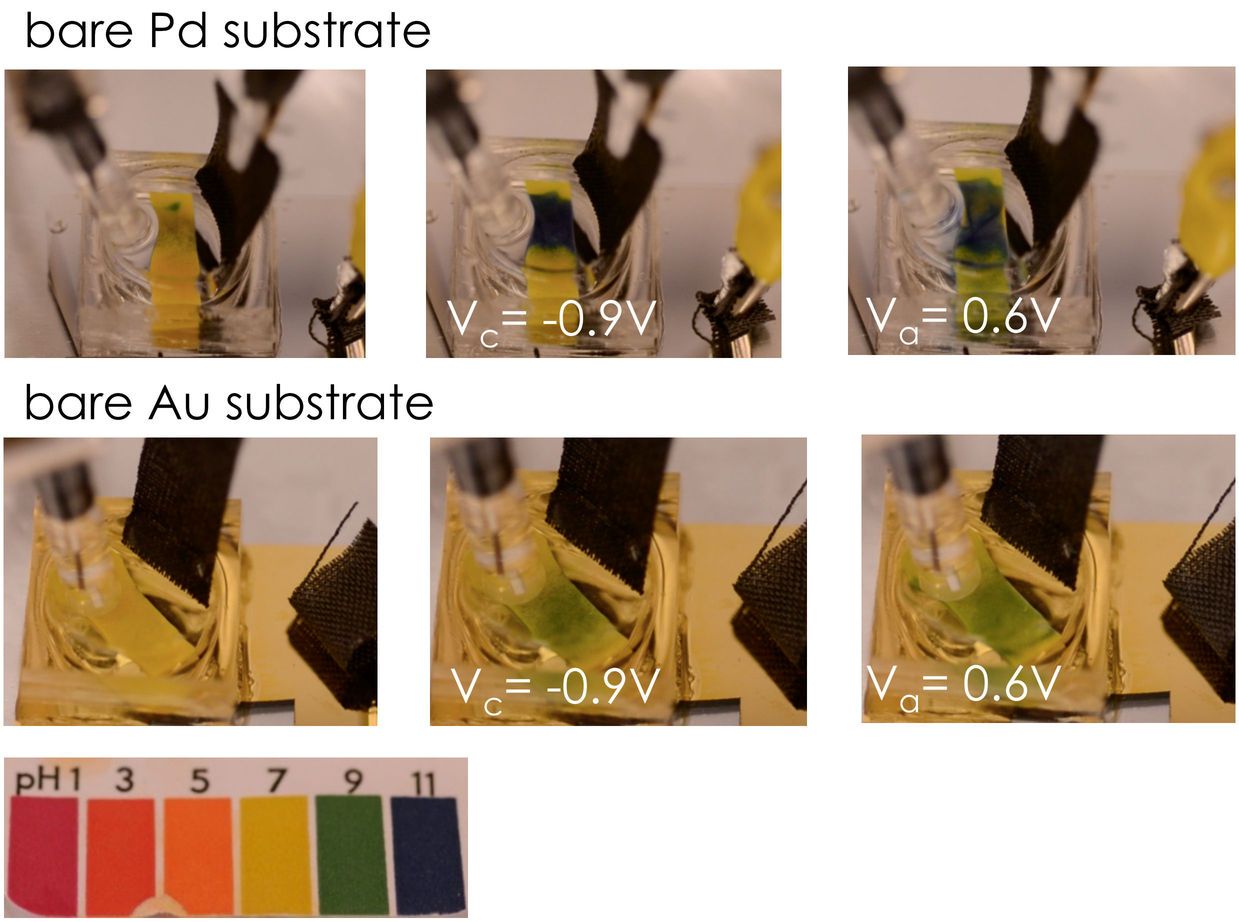

Fig. S3. pH modulator with Pd working electrode and Au electrode. A pH stripe is in the PDMS chamber to show the pH change.

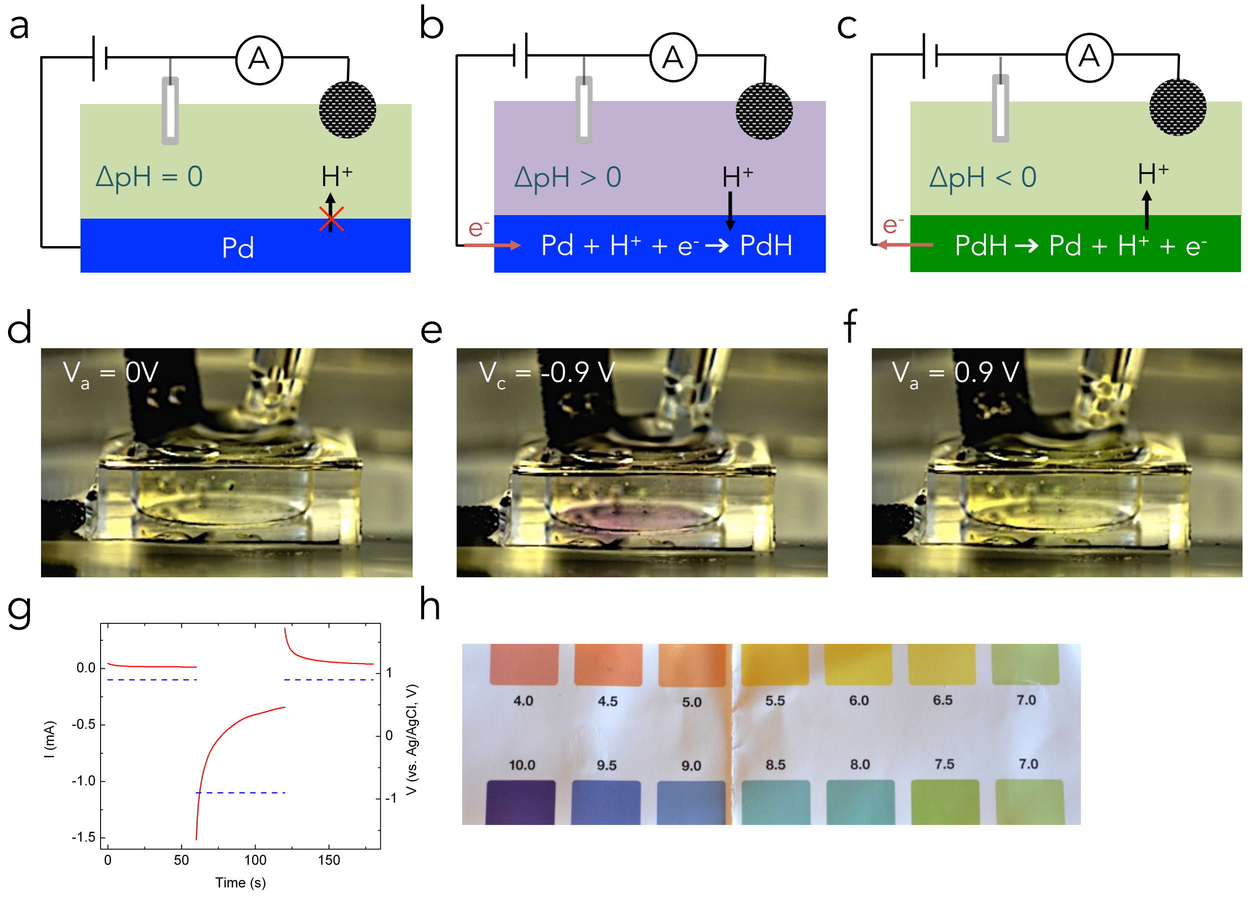

Fig. S4. pH regulation in solution. (a).(b).(c). Schematics of pH modulator with CNT modified CF counter electrode. In (a), with Pd contact there is no I_a at V_a = 0.9 V. (b). At V_c = -0.9 V, H+ transfers from solution into the Pd and recombines with e- to form H. H adsorbs onto Pd and forms PdH. Decrease of H+ concentration in solution causes pH increase in solution. (c). V_a = 0.9 V causes H+ transfer from PdH into solution, resulting in pH decrease. (d). (e). (f). Pictures of pH modulator with pH indicator in solution. (d) corresponds to (a). Green color indicates pH= 7.0 in solution, which is the initial pH of solution. (e) corresponds to (b). Purple color indicates pH = 10.0 in solution. pH increase is due to H+ transfer from solution to Pd contact. (f) corresponds to (c). The solution returns to the initial green color, showing the pH changes from basic to neutral. (g). I-V curve corresponding to time. (h) Color scale of pH indicator.

Voltage	Initial pH	Final pH	H^+ change (C)	I*Time (C)
V_c = -1.2V	6.8	8.4	-1.54e-5	-0.04
V_a = 0V	8.4	6.2	3.34e-5	0.11
V_c = -1.2V	6.2	9.2	-6.06e-5	-0.15
V_a = 0V	9.2	6.7	1.92e-5	0.07
V_c = -1.2V	6.7	9.3	-1.92e-5	-0.17
V_a = 0V	9.3	6.2	6.06e-5	0.03

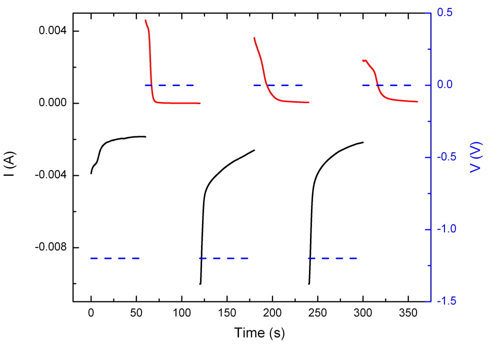

Fig. S5. pH change with the pH modulator when applied V_c and V_a. The pH value is measured with a pH meter. The pH change repeats 3 cycles. Total charge from the pH change is compared to the total charge from the integration of current I by Time.

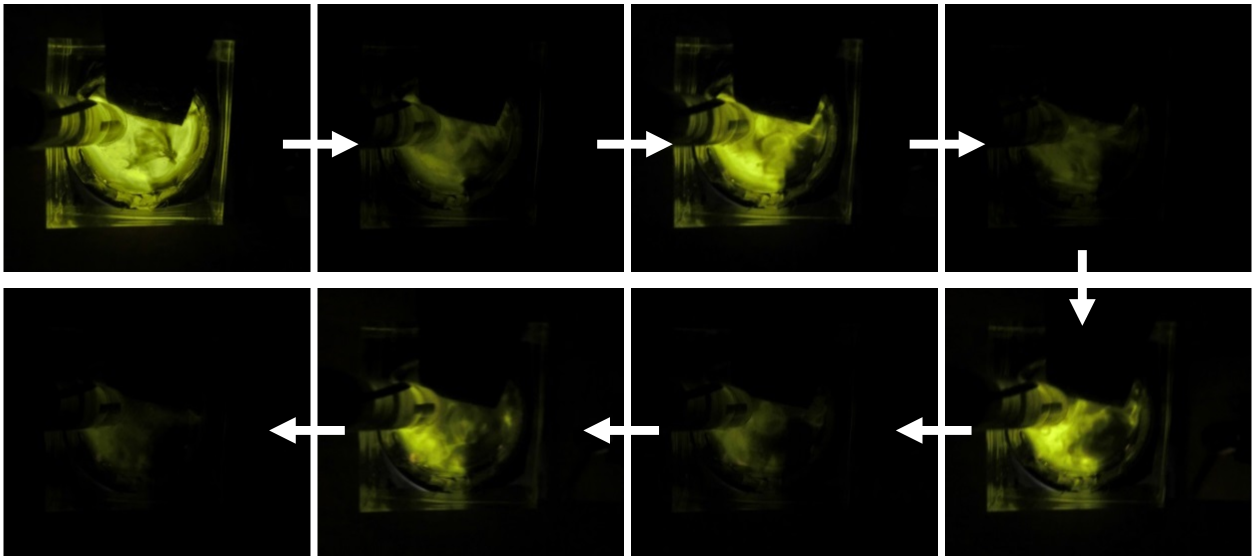

Fig. S6. Four cycles of bioluminescence turned on and off by the pH modulator
